# Predictors of catastrophic out-of-pocket health expenditure in rural Egypt: application of the heteroskedastic probit model

**DOI:** 10.1186/s42506-021-00086-x

**Published:** 2021-08-03

**Authors:** Suzan Abdel-Rahman, Farouk Shoaeb, Mohamed Naguib Abdel Fattah, Mohamed R. Abonazel

**Affiliations:** 1 Department of Biostatistics and Demography, Faculty of Graduate Studies for Statistical Research, Cairo University, Giza, Egypt; 2 Department of Applied Statistics and Econometrics, Faculty of Graduate Studies for Statistical Research, Cairo University, Giza, Egypt

**Keywords:** Out-of-pocket health payments, Catastrophic health expenditure, Multiplicative heteroskedastic probit model

## Abstract

**Background:**

Out-of-pocket (OOP) health expenditure is a pressing issue in Egypt and far exceeds half of Egypt’s total health spending, threatening the economic viability, and long-term sustainability of Egyptian households. Targeting households at risk of catastrophic health payments based on their characteristics is an obvious pathway to mitigate the impoverishing impacts of OOP health payments on livelihoods. This study was conducted to identify the risk factors of incurring catastrophic health payments hoping to formulate appropriate policies to protect households against financial catastrophes.

**Methods:**

Using data derived from the Egyptian Household Income, Expenditure, and Consumption Survey (HIECS), a multiplicative heteroskedastic probit model is applied to account for heteroskedasticity and avoid biased and inconsistent estimates.

**Results:**

Accounting for heteroskedasticity induces notable differences in marginal effects and demonstrates that the impact of some core variables is underestimated and insignificant and in the opposite direction in the homoscedastic probit model. Moreover, our results demonstrate the principal factors besides health status and socioeconomic characteristics responsible for incurring catastrophic health expenditure, such as the use of health services provided by the private sector, which has a dramatic effect on encountering catastrophic health payments.

**Conclusions:**

The marked differences between estimates of probit and heteroskedastic probit models emphasize the importance of investigating homoscedasticity assumption to avoid policies based on incorrect evidence. Many policies can be built upon our findings, such as enhancing the role of social health insurances in rural areas, expanding health coverage for poor households and chronically ill household heads, and providing adequate financial coverage for households with a high proportion of elderly, sick members, and females. Also, there is an urgent need to limit OOP health payments absorbed by private sector to achieve an acceptable level of fair financing.

## Introduction

Diseases could cause catastrophic health payments, undermine the process of income generation, and jeopardize future economic welfare [1]. Catastrophic health expenditure (CHE) occurs when out-of-pocket (OOP) health payments absorb a large portion of the resources of a household to the extent that they severely affect the household’s living standard [2]. In the short term, catastrophic payments disrupt household consumption of other essential needs, whereas, in the long term, they may force households to deplete assets, use savings, sell livestock, and incur debts [3–5].

Substantial health expenditures push households into poverty or deeper [6, 7]. Approximately 30% of households in 15 African countries had to borrow and sell assets to finance health payments; this percentage increased to 50% when facing hospitalization expenses and to 40% when using other health services [8]. Even modest health payments cause indebtedness with exorbitant interest rates besides the sale of consumables and assets, which ultimately lead to poverty [9].

Two approaches are commonly applied to measure the incidence of catastrophic health payments: in the first approach, catastrophic health payments are defined as health expenditures exceeding a specific fraction of the total household expenditure or nonfood expenditures [10]. In the second approach, they are defined as the ability to pay, which is measured after deducting subsistence spending [6]. Both approaches asserted that OOP health payments accelerate and exacerbate poverty; coping strategies with health payments create a vicious circle of indebtedness and impoverishment, and must be considered to understand the trend and impetus of poverty over time [11].

Although public health investments dwindle annually in Egypt, urban areas have captured the largest percentage of public health spending, whereas rural areas have invariably received less attention and are poorly funded. The gap between health spending in urban and that in rural areas has reached roughly 67%, which is reflected in a substantial disparity in the availability of healthcare infrastructure. Moreover, rural areas are characterized by low levels of income, education, and economic development. All these factors may contribute to the high incidence rates of catastrophic payments [12].

This study was conducted to estimate the incidence of CHE, identify its core determinants, and reveal the extent to which health insurance schemes properly target vulnerable economic households and whether they succeed in protecting them financially. This study contributes to the literature by exploring the predictors of CHE using a multiplicative heteroskedastic probit model, that accounts for heteroskedasticity and provides unbiased and consistent estimates [13]. Moreover, this study used the latest round of the Egyptian Household Income, Expenditure, and Consumption Survey (HIECS), which enables us to incorporate many fundamental covariates neglected by other studies, including the type of health insurance, health service providers, and household composition [12].

## Methods

### Data

Data were obtained from the Egyptian Household Income, Expenditure and Consumption Survey) HIECS), a nationally representative survey conducted by Central Agency for Public Mobilization and Statistics) CAPMAS) in 2015 [14]. The HIECS uses three different types of questionnaires: Expenditure and Consumption Questionnaire, Assisting Questionnaire, and Income Questionnaire. The survey collects data on demographic, socioeconomic, and health characteristics of rural and urban households, selected using a two-stage stratified random sampling design. HIECS measures consumption patterns and provides detailed information on household’s income sources and health status. Individuals were asked about suffering any illness during 6 months preceding the survey, having a chronic disease or a disability, healthcare providers, and health insurance coverage and its type.

Out-of-pocket healthcare expenditures (OOP) comprise inpatient and outpatient health services and other reported expenditures (medicine, laboratory tests, X-rays, medical equipment) and do not include insurance premiums. We used household as the unit of analysis in order to account for the intra-household distribution of resources and coping strategies with health payments, assuming that the economic impact of diseases affects all household members. Our analysis focuses on rural areas including 6670 households. Rural households are distributed mainly in Rural Lower Egypt and Rural Upper Egypt. All estimates are adjusted to represent national figures using appropriate sampling weights. Both descriptive and analytical statistics were performed using R software.

### Measuring incidence of catastrophic OOP health payment

Catastrophic health expenditure was defined as “Any health expenditure that threatens a household’s financial capacity to maintain its subsistence needs and does not necessarily equate to high healthcare costs. Even relatively small expenditures on health can be financially disastrous for poor households” [2]. According to the methodology proposed by Xu et al. [6], the most common approach applied in recent studies, OOP health expenditure is catastrophic whenever it is greater than or equal to 40 percent of the household’s capacity to pay, where:

- Capacity to pay (*ctp*_*h*_) is the remained household income after satisfying food subsistence spending.
- Subsistence spending (*se*_*h*_) is determined based on average food expenditures of households with food share within the 45th and 55th percentile of the total sample. Actual food expenditure is used to measure the capacity to pay for the household whose food expenditure is less than subsistence spending. Expenditures are adjusted using the economy of scale estimated by Xu et al. (2003) (*β*=0.56) to reflect food consumption sharing among household members. Xu’s methodology has been clearly described elsewhere [6].

### Multiplicative heteroskedastic probit model

Misspecification in limited dependent models that may result from heteroskedasticity, omitted variables, heterogeneity and nonlinearity leads to inconsistent and biased estimates. The quasi maximum likelihood estimator (QMLE) of the probit model is inconsistent in case of violation of homoscedasticity, even using white corrected estimator for standard errors is useless in the heteroskedastic probit model because the QMLE itself is biased [13].

Let *c*_*i*_ denote a binary response (0,1) representing the occurrence of catastrophic health expenditure (CHE) when household health expenditure (*h*_*i*_) exceed its capacity to pay (*ctp*_*i*_). The subscript *i* denotes the household:

1 $$ {c}_i=\left\{\begin{array}{c}1\kern3em {h}_i>{ctp}_i\\ {}0\kern3.25em {h}_i\le {ctp}_i\end{array}\right. $$

The probability of incurring catastrophic health expenditure,

2 $$ \Pr \left({c}_i=1\right)=\Pr \left({\varepsilon}_i<{y}_i^{\prime}\beta \right)=\Phi \left({y}_i^{\prime}\beta \right) $$

*y*_*i*_is a *k* × 1vector of observed independent variables(*y*_1*i*_, *y*_2*i*_, *y*_3*i*_…. *y*_*ki*_), *β* is vector of the corresponding parameters, *ε*_*i*_ is random error term and Φ(.) is the cumulative distribution function of standard normal variable with mean 0 and variance 1.

Total household consumption is a superior proxy for household economic status; it is less subject to fluctuations and measurement errors than income. Also, it includes the value of home production, which is a major source of income in developing countries, especially rural areas [15]. Consequently, household consumption was used to avoid underestimating the living standards of rural households whose members were engaged in the agriculture sector and exhibited varied seasonal incomes.

The probit model assumes that the error distribution of the latent model is homoscedastic and has unit variance. However, many studies that examined the determinants of catastrophic health payments have neglected to verify the violation of the homoscedasticity assumption, which could result in substantially biased and inconsistent estimates besides misspecified standard errors. Specific covariates may influence the probability of incurring catastrophic payments through health payment variance. On average, better-off households may spend more on healthcare services exposing them to CHE, but also the large variance of health payments at high expenditure levels due to uncommon and sophisticated medical treatments will raise the incidence of CHE. This variance is hypothesized to increase with household expenditure as poor households have constrained budgets limiting their response to health shocks, whereas rich households that face illness episodes spend too much compared with rich households with good health. A heteroskedastic probit model is applied to consider that health expenditure *h*_*i*_ is heteroskedastic with unfixed variance${\sigma}_i^2$. A heteroskedastic probit model generalizes Φ(.) to a normal cumulative distribution function (CDF), allowing variance to vary systematically as a multiplicative function of set of *m* explanatory variables *x*_*i*_ [13, 16].

3 $$ {\sigma}_i^2={\left\{\exp \left({x}_i^{\prime}\tau \right)\right\}}^2 $$

where *x*_*i*_ is *m* × 1 vector of covariates (*x*_1*i*_, *x*_2*i*_, *x*_3*i*_, .. *x*_*mi*_) that determine the variance of the error term, while *τ* is the corresponding coefficient vector.

Therefore, the probability of incurring catastrophic health payments is

4 $$ \Pr \left({c}_i=1\right)=\Phi \left\{\frac{y_i^{\prime}\beta }{\exp \left({x}_i^{\prime}\tau \right)}\right\} $$

A multiplicative heteroskedastic probit model relaxes the homoscedasticity by allowing the scale of the inverse link function to vary systematically from one observation to another as a function of explanatory variables. It is fitted via maximum likelihood where the log-likelihood function takes the form:

5 $$ lnL=\sum \limits_{i\in F}{h}_i\ \mathit{\ln}\Phi \left\{\frac{y_i^{\prime}\beta }{\exp \left({x}_i^{\prime}\tau \right)}\right\}+\sum \limits_{i\notin F}{h}_i\ \mathit{\ln}\left[1-\Phi \left\{\frac{y_i^{\prime}\beta }{\exp \left({x}_i^{\prime}\tau \right)}\right\}\right] $$

where *F* is the set of all observations *i* such that *c*_*i*_ ≠ 0 and *h*_*i*_denote the weights.

All explanatory variables enter *y* vector while total expenditure variable is included in *x*_*i*_ vector for modelling the heteroskedastic variance of the error term${\sigma}_i^2$. The marginal effects are more informative and directly interpretable than multiplicative effects (probit model’s coefficients). The heteroskedastic probit model’s marginal effects are estimated as a function of the parameters *β* and *τ* [13] as follow:

The marginal effect of continuous variable *w*_*z*_ is

6 $$ \frac{\partial \Pr \left({c}_i=1\right)}{\partial {w}_z}=\phi \left(\frac{y_i^{\prime}\beta }{\exp \left({x}_i^{\prime}\tau \right)}\right)\frac{\beta_z-{y}_i^{\prime}\beta .{g}_z}{\exp \left({x}_i^{\prime}\tau \right)} $$

where *ϕ*(.) is the probability density function (pdf). If the variable *w*_*z*_ is an element of both *y* and *x* vectors, its coefficient in the mean equation (1) is *β*_*z*_ and in the variance equation (4) is *g*_*z*_, while *g*_*z*_ equals 0 if *w*_*z*_ appears only in *y* vector.

The marginal effect of dummy variable *w*_*z*_ is

7 $$ \frac{\partial \Pr \left({c}_i=1\right)}{\partial {w}_z}=\Phi \left(\frac{y_1^{\prime}\beta }{\exp \left({x}_1^{\prime}\tau \right)}\right)-\Phi \left(\frac{y_0^{\prime}\beta }{\exp \left({x}_0^{\prime}\tau \right)}\right) $$

*w*_*z*_ is set to 1 in *y*_1_ and *x*_1_ and set 0 in *y*_0_ and *x*_0_. Standard errors of the marginal effects are derived to allow inference and hypothesis testing, using delta method which is the most commonly used [17].

To investigate the determinants of CHE, dummy variables for the characteristics of household heads were included in the analysis, such as gender, illiteracy status, and education level. Besides, the analysis comprised age and squared age of the household heads to control for the nonlinearity of age. Several household-level covariates were also incorporated, such as the presence of chronic diseases and disability; health insurance coverage; and proportions of sick members, insured members, insured children, insured elderly, wage earners, and educated members, in addition to household total expenditure and geographic area. To capture the effects of household composition, we included household size and the proportions of children, elderly, and females.

## Results

The distribution of annual health expenditure was right-skewed (skewness = 7.27), the mean annual health expenditure (LE 2903.4) was higher than the median (LE 1706.3). The positive excess kurtosis (101) indicated that health expenditure distribution is leptokurtic. After taking the log transformation, the mean value became 7.316, whereas the median value was 7.42, skewness was 0.27, and kurtosis was 1.4. The average annual household income was LE 37625. The average household size was five members, with 25.6% being males. One of five rural households was headed by a female. The age composition of the households indicated that 28.2% of household members were children and 8.62% are elderly; nearly 18.2% of the households had at least one elderly member. In respect to household head characteristics, 66.2% of household heads were literate, approximately half of them had at least an elementary certificate, only 0.8% had higher university degrees, 77.7% were employed, 73.5% had chronic diseases, and only 31.7% were insured.

**Fig. 1:**
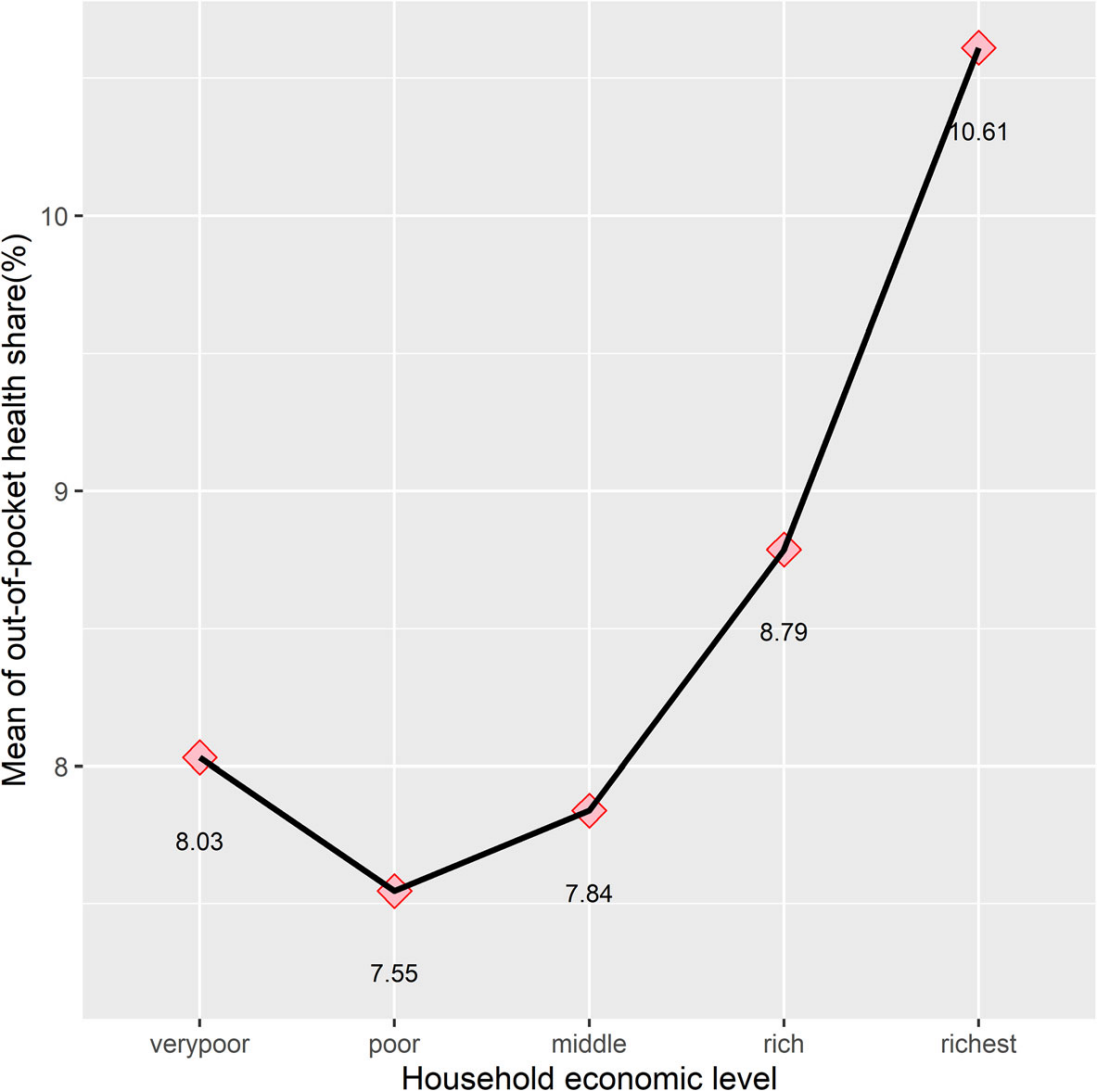
The mean out-of-pocket health share by economic level

The average OOP payments were significantly higher among better-off households than those among poor ones. The richest households spend nearly five times that of what the poorest households spend on healthcare services (LE 6954.28 vs. LE 1483.5). Meanwhile, the health share of the total budget ranged from 8.17% at the poorest economic level to 11.08% at the richest level, indicating that poor households cannot deduct a large percentage of their budget for healthcare services. The health share of the household budget has an upward trend with the economic level (Fig. 1). Disaggregated health expenditures enable us to distinguish between budget shares allocated to inpatient and outpatient care across economic levels. The results showed that the expenditure share on inpatient services in rich households was nearly four times that in poor households (2.55% vs. 0.6%), whereas both poor and rich households allocated similar shares of their budgets to drugs (4.4% and 4.7%, respectively).

Egypt has a narrow geographic distribution of health services, which is more concentrated in urban areas. Households across the socioeconomic strata in urban areas witness extensive healthcare infrastructure in contrast to rural households, which resulted in heavy reliance on the purchasing of non-prescribed drugs in rural areas. Drug purchases account for a larger share of OOP health payments of rural households (54.7%), followed by outpatient health services (38.7%), whereas inpatient services acquire the smallest share (6.6%).

An investigation of the components of health expenditure across various economic levels indicated the overuse of medicines among rural households, particularly poor households; pharmaceutical products had the largest share of OOP health expenditure in the poorest level compared with other quintiles (62.25% in the poorest level vs. 48.1% in the richest level). Meanwhile, inpatient services account for 11.97% of the total health expenditure of rich households compared with only 4% of the total health expenditure of poor households.

**Fig. 2:**
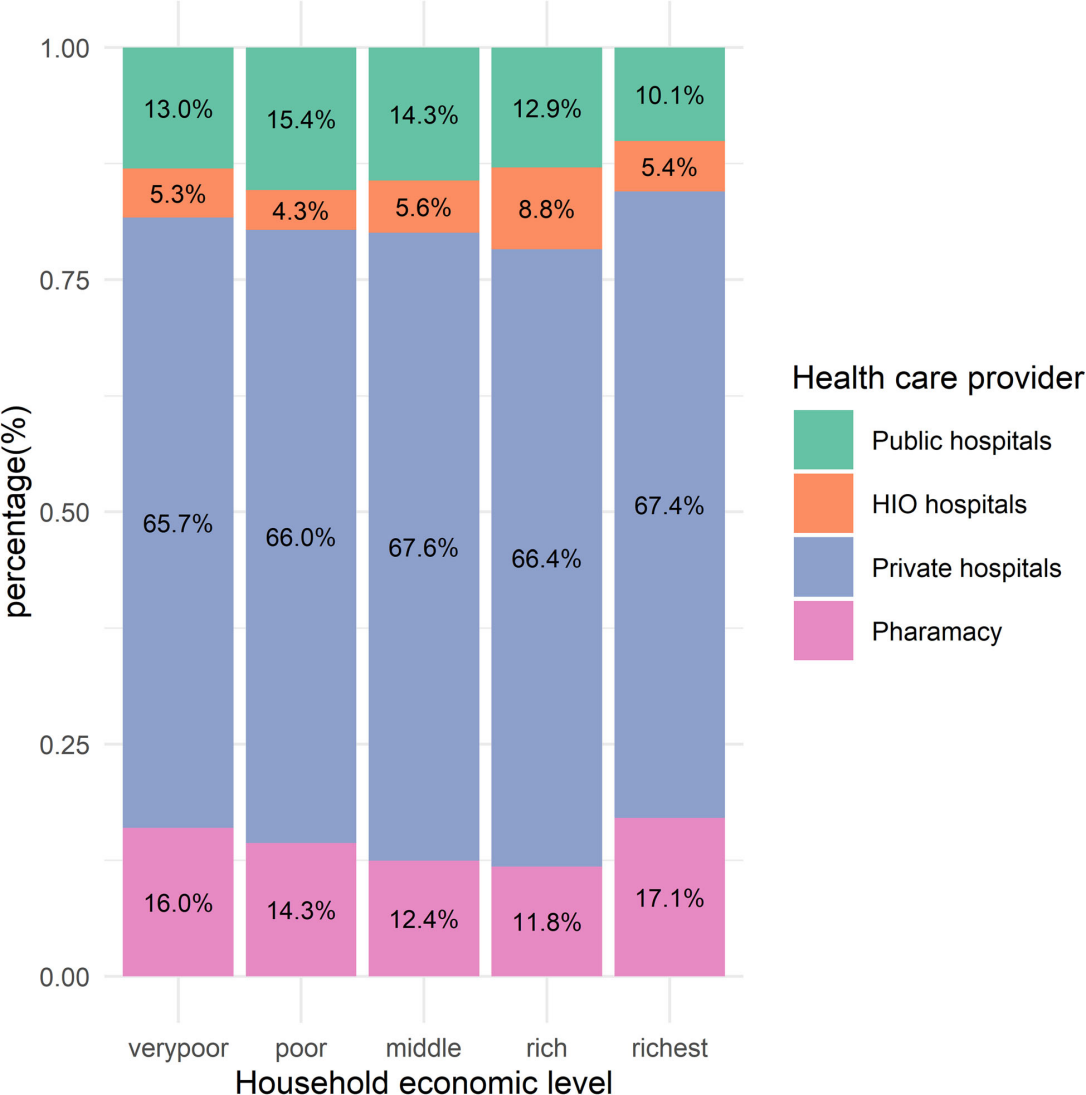
Distribution of rural households by economic level and healthcare provider

The amount of OOP health payments depends mainly on where households seek healthcare. The data revealed a significant variation in the paid health shares by the type of healthcare providers; rural households using private hospitals and clinics paid on average 12.92% of their budgets on healthcare services. Also, households seeking healthcare in public hospitals and social health insurance hospitals allocate a high share, accounting for 10.5% and 10%, respectively, of their total budget. Households that depend on medical consultations from pharmacies allocate 9.48% of their budgets. However, civil society health services were associated with a relatively small share (6.19%). Although private healthcare providers demand higher payments than public healthcare providers and are hypothesized to be less used by households at low economic levels, the data indicated that, contrary to expectations, private healthcare providers are the most used across all economic levels; 65.58% of the poorest households use private hospitals and clinics, and similar proportions were found at other economic levels (Fig. 2).

**Fig. 3:**
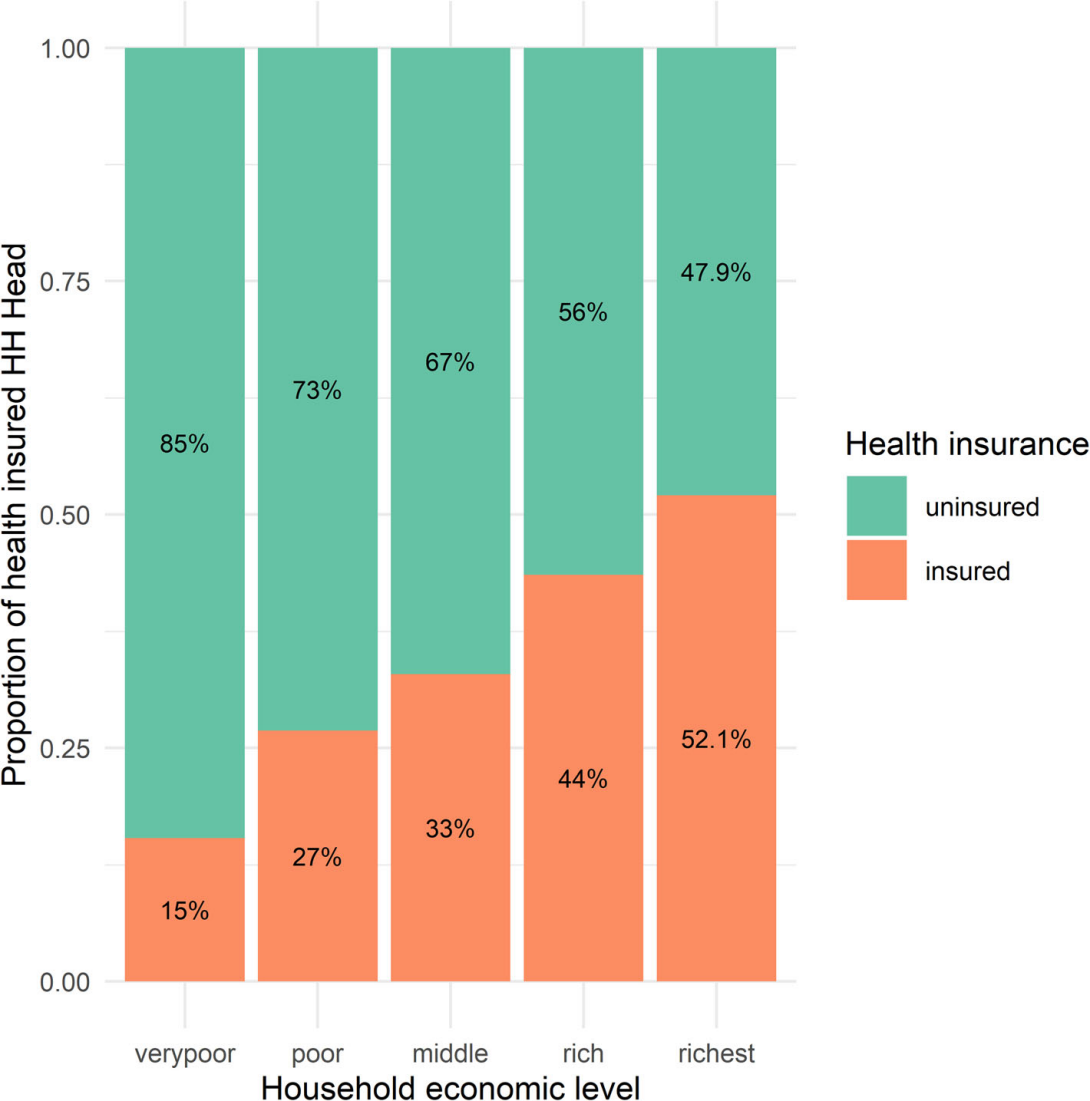
Distribution of rural households by health insurance coverage of household head across economic levels

However, risk-pooling mechanisms are significant tools for protecting vulnerable households from unexpected health risks. The HIECS indicates that health insurance coverage is low in rural areas. Nearly 68.25% of household heads are uninsured, even though 71.64% of them have at least one chronic disease. The high proportion of informal workers, which characterizes rural areas, explains the low health insurance coverage for household heads. Additionally, apparent inequalities exist in health insurance coverage across genders, education levels of household heads, and other socioeconomic characteristics of households. The proportion of insured male household heads is three times that of their female counterparts (36% vs. 12%(; the proportion of insured household heads increases sharply as the household economic level increases, rising from 15% in the poorest level to 52.2% in the richest level (Fig. 3). Meanwhile, at the household level, on average, half of the household members are insured, which is due to the compulsory insurance for children in schools where the average number of insured children in the household is 3, whereas, on average, only one elderly member in the household is insured.

**Fig. 4:**
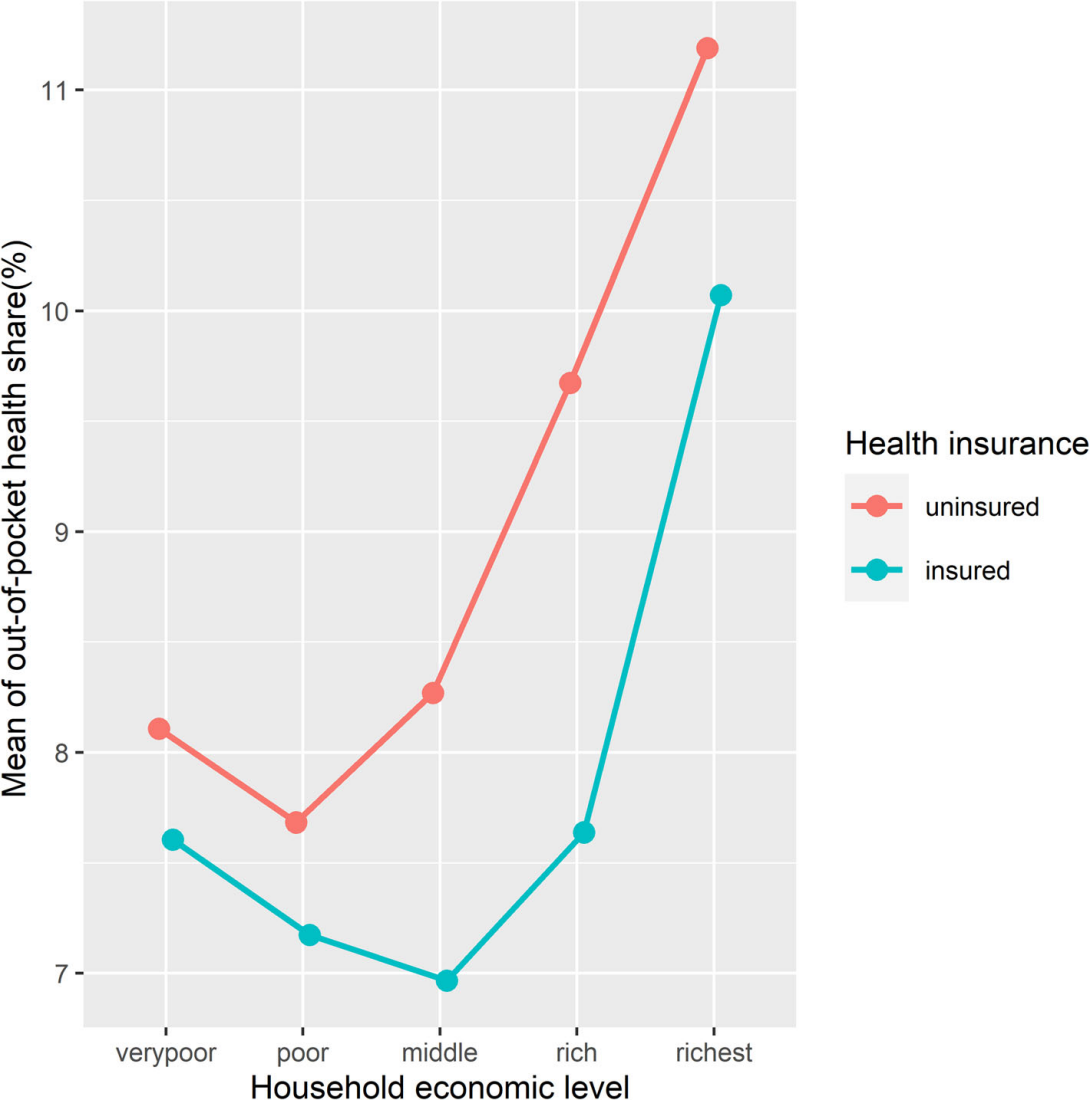
The mean out-of-pocket health share by economic level and health insurance status of household heads

A significant difference in the budget share allocated to health was observed between insured and uninsured households. Although uninsured households spend, on average, less than insured households (LE 2827.6 vs. LE 3426.9), they allocate larger shares to health spending (8.8% vs. 8.3%). Also, health shares of households with uninsured heads were significantly higher than those of households with insured heads across all economic levels (Fig. 4). A two-way analysis of variance (ANOVA) was performed to simultaneously assess the effects of both health insurance coverage and economic level on health expenditure and revealed that both variables have significant effects on health expenditure at a significance level of 0.01.

A comparison of health expenditure on inpatient and outpatient services across insured and uninsured households revealed the languid role of health insurance in protecting rural households against high health costs. Outpatient care expenditures among insured households are significantly higher than those among uninsured households; insured households spend, on average, approximately LE 1422.3 on outpatient healthcare services compared with LE 888.97 by uninsured households (3.58% vs. 2.79% of household budget). To simultaneously test whether expenditures on both inpatient and outpatient services vary between insured and uninsured households, multivariate analysis of variance (MANOVA) was performed. The global multivariate test was significant and indicated that the effect of health insurance coverage was significant with a Pillai test statistic of 0.03 and a *p* value of below 0.001.

**Fig. 5:**
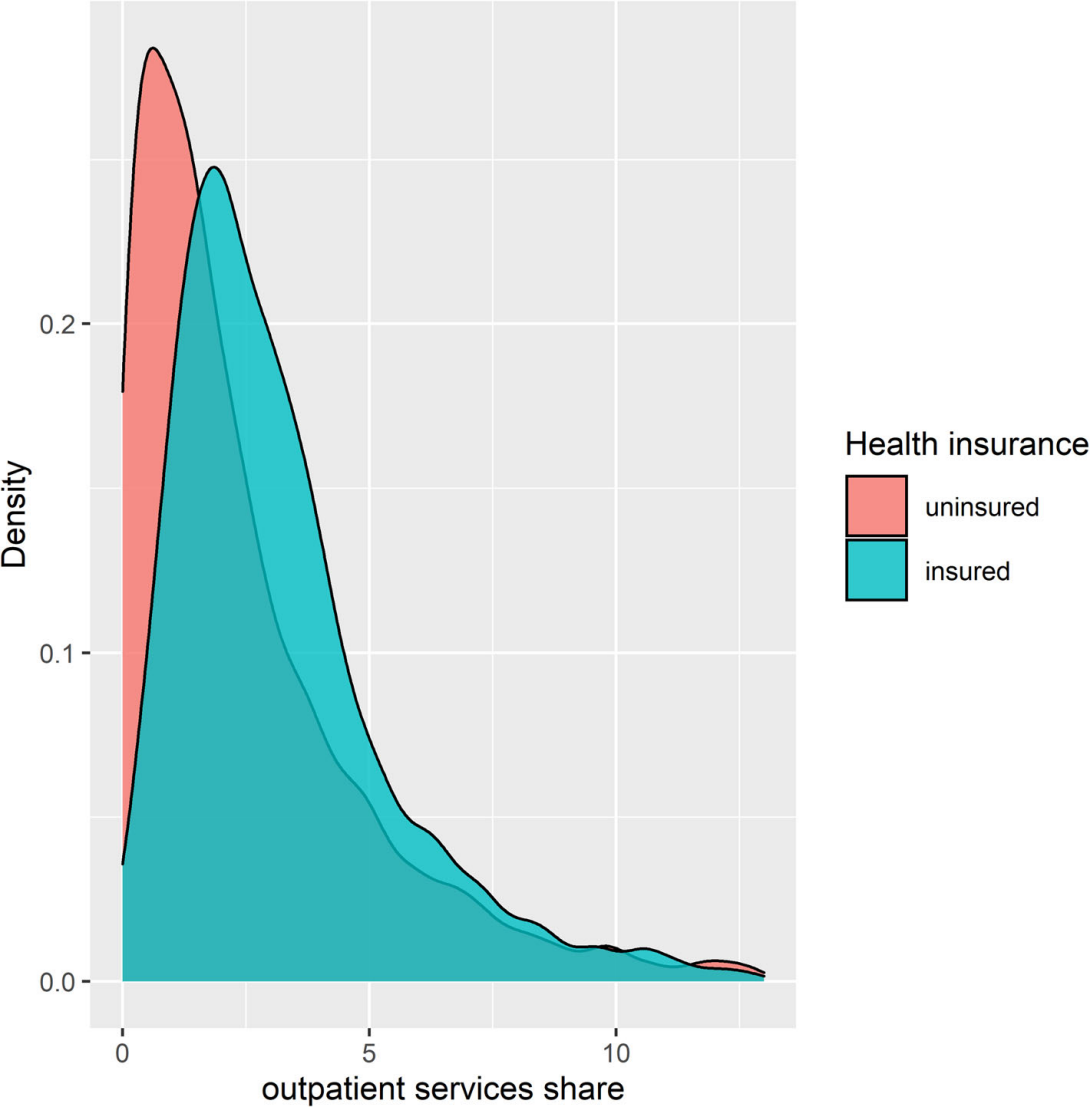
Distribution of outpatient services share across health insurance status

Also, the share distribution for outpatient services highlights the relatively larger shares incurred by insured households (Fig. 5). A different conclusion is drawn for the effect of insurance coverage on pharmaceutical spending; the pharmaceutical share spent by insured households is significantly less than that incurred by uninsured households (4.61% vs. 4.86%).

**Fig. 6:**
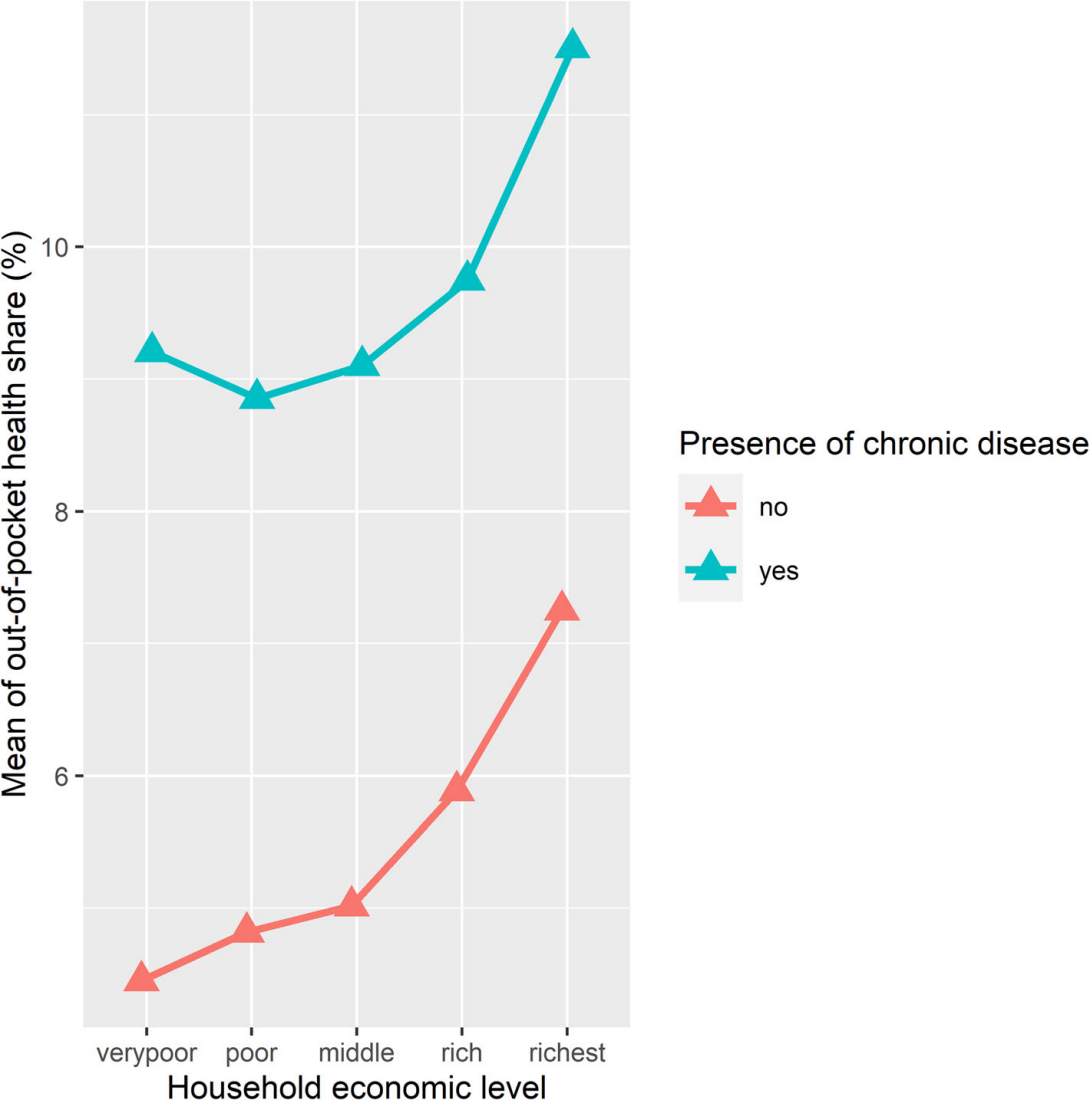
The mean out-of-pocket health share according to the presence of chronic diseases

Data revealed that rural areas have a high prevalence rate of chronic diseases; every rural household has at least one member with chronic disease, and the average number of household members with chronic diseases is 3. Households suffering from chronic diseases spend more than twice that of what households without chronic diseases spend. The health share of households experiencing chronic diseases has a clear increasing pattern with the economic level (Fig. 6).

The incidence of CHE is small in rural households as a whole (7.91%), but large among the poorest households (14.59%). The incidence of CHE has regressive distribution across economic quintiles: it declines sharply from 14.59% in the poorest quintile to 5.93% in the poor quintile, and it reaches 5.26% and 4.92% among households in the middle and rich economic levels, respectively; then it rises to 7.81% among the richest households. The presence of chronic diseases significantly increases the incidence of CHE (*p* < 0.01) and makes the pattern more serious at some economic levels. At the lowest economic level, the incidence rate of CHE among households with chronic diseases is 16.22% compared with 9.46% among households without chronic diseases. While a better economic status decreases the catastrophic payment gap between households with and without chronic diseases, the presence of chronic diseases increases the overall incidence rate of CHE from 7.88 to 9.18%. Our data indicated also that having health insurance decreases the incidence of CHE among households with chronic diseases to 5.44% compared with 10.90% among households without health insurance coverage.

A homoscedastic probit model was used along with a multiplicative heteroskedastic probit model in which the total household expenditure becomes a part of the scale sub-model. The results of both models are provided in Table 1. The likelihood ratio test of heteroskedasticity substantiates a violation of homoscedasticity; the total household expenditure is more correlated with variance. Coefficients are significant and have the hypothesized signs in the mean function of the heteroskedastic probit model. The likelihood ratio test on the nested models prefers the heteroskedastic probit model over the ordinary probit model. Moreover, comparing the two models using information metrics also confirmed that the heteroskedastic probit model is the better model as it has lower Akaike’s information criteria. Also, the Bayesian information criteria that strongly penalizes including additional variables to the model demonstrates the same conclusion.

**Table 1:** Estimates of homoscedastic probit model and heteroskedastic probit model, HIECS, 2015

Variables	Probit model		Heteroskedastic probit model	
	Coef.	Marginal effect	Coef. "Mean model"	Marginal effect
**Intercept**	− 2.407*** (0.504)		− 2.398* (1.239)	
**Male-headed household**	0.363*** (0.087)	0.045*** (0.009)	0.816*** (0.204)	0.061** (0.053)
**Age of household head**	− 0.012 (0.0127)	− 0.001 (0.002)	− 0.010 (0.026)	− 0.001 (0.001)
**Squared age of household head**	0.000 (0.000)	0.000 (0.000)	− 0.010 (0.026)	0.001 (0.001)
**Illiterate household head**	0.126* (0.071)	0.018* (0.010)	0.239 (0.159)	0.049** (0.071)
**Educational degree of household head (Dummy variables)**				
Having a primary or lower secondary degree Having a secondary or post-secondary degree Having a university degree Having a postgraduate degree None (omitted category)	− 0.196 (0.091) − 0.151* (0.091) − 0.469** (0.154) − 0.028 (0.474)	− 0.015 (0.011) − 0.019* (0.010) − 0.048*** (0.011) − 0.003 (0.063)	− 0.151 (0.212) − 0.352* (0.199) − 1.273** (0.182) − 1.311 (2.176)	− 0.013 (0.023) − 0.053* (0.170) − 0.094*** (0.021) − 0.083* (0.063)
**Employed household head**	− 0.255*** (0.474)	− 0.038** (0.012)	− 0.528** (0.162)	− 0.072** (0.069)
**Geographical area:** rural lower Egypt	0.722* (0.367)	0.126* (0.065)	1.868* (1.003)	0.321** (0.091)
**Household size**	− 0.0206 (0.027)	− 0.002 (0.003)	0.158** (0.060)	0.032** (0.021)
**Proportion of females**	0.437*** (0.132)	0.059*** (0.018)	0.592** (0.283)	0.082** (0.031)
**Proportion of children in household**	0.186 (0.169)	0.025 (0.023)	0.297 (0.397)	0.064 (0.317)
**Proportion of elderly in household**	0.271* (0.135)	0.037* (0.018)	0.200** (0.260)	0.029** (0.012)
**Presence of chronic diseases**	0.324*** (0.064)	0.039*** (0.007)	0.769*** (0.152)	0.097*** (0.032)
**Presence of disability**	0.164 (0.012)	0.025 (0.021)	0.118 (0.261)	0.101 (0.073)
**Proportion of sick members in household**	0.218* (0.19)	0.031* (0.017)	0.506* (0.269)	0.063** (0.072)
**Presence of insurance coverage**	− 0.213*** (0.064)	− 0.027*** (0.007)	− 0.447** (0.159)	− 0.042*** (0.029)
**Household total expenditure** ^**a**^	− 0.003 (0.002)	− 0.009 (0.007)	− 0.167*** (0.012)	− 0.071*** (0.47)
**Proportion of wage earners in household**	− 0.219 (0.178)	− 0.027 (0.022)	− 4.052** (0.495)	− 0.271*** (0.149)
**Proportion of educated members in household**	− 0.551*** (0.164)	− 0.066*** (0.019)	− 1.373** (0.473)	− 0.332*** (0.017)
**Proportion of children covered by Health Insurance**	− 0.355 (0.266)	− 0.039 (0.029)	− 1.595 (1.014)	− 0.071 (0.017)
**Proportion of elderly covered by Health Insurance**	0.238 (0.912)	0.035 (0.721)	0.934 (1.127)	0.011 (0.23)
**Type of health insurance coverage (Dummy**				
**variables)** Covered by HIO	− 0.028 (0.174)	− 0.004 (0.023)	− 0.229 (0.423)	− 0.061 (0.093)
Covered by private insurance	0.391 (0.433)	0.068 0.093)	0.617 (1.02)	0.252 (0.093)
Covered by employer-provided private insurance	− 0.279 (0.259)	− 0.031 (0.024)	− 0.594 (0.656)	− 0.081 (0.074)
Covered by occupational syndicates	0.391 (0.284)	0.068 (0.061)	1.285* (0.684)	0.168* (0.051)
Other (omitted category)				
**Health provider (Dummy variables)**				
HIO hospitals	4.793** (0.169)	0.027 (0.028)	0.161* (0.400)	0.012* (0.021)
Public hospitals	4.275*** (0.101)	0.085 (0.017)	0.153* (0.224)	0.031* (0.021)
Private hospitals	0.531*** (0.064)	0.066*** (0.009)	1.097* (0.146)	0.389** (0.071)
Pharmacy	− 0.081 (0.124)	− 0.026 (0.012)	− 0.418 (0.245)	− 0.076 (0.032)
Other(omitted category)				
Using outpatient health services	0.131 (0.101)	0.019 (0.017)	0.049* (0.242)	0.091* (0.021)
Het-test ***χ***^**2**^	------		52.24***	
LogL	− 1566.968		− 1541	
Likelihood ratio test	229.13***			
McFadden’s pseudo ***R***^**2**^	0.069			
AIC	3175.94		3125.69	
BIC	3317.12		3273.603	
Number of fisher scoring iterations	6		10	
*N*	6143		6143	

***p<0.001, **p<0.01, *p<0.05, ( ): robust standard errors against misspecification.

^a^Coefficient and standard error of household total expenditure in latent scale model is{0.003*** (0.002)}.

LogL is the maximum value of the likelihood function.

Likelihood ratio test measures the significance of the overall model.

Pseudo ***R***^**2**^shows the amount of variance explained by explanatory variables.

Wald *χ*^2^ test the significance of the full model; if all slopes equal 0.

Het-test *χ*^2^ is the likelihood ratio test of the null hypothesis of homoscedasticity; it tests the model with heteroskedasticity against the full model with out.

AIC and BIC are information metrics that penalize the inclusion of additional variables and used to select the appropriate model.

Number of fisher scoring iterations indicates how quickly the iterative weighted least squares (IWLS) algorithm terminates.

Significant differences in coefficients and marginal effects were observed between the probit and heteroprobit models, emphasizing how large the estimates are biased in the ordinary model. First, the impact of most variables increases dramatically in the heteroskedastic probit model, such as the effects of proportions of wage earners, educated members, and insured children in the household, whereas the impact of some variables diminishes, such as the type of healthcare provider (e.g., public hospitals and hospitals affiliated with the health insurance organization (HIO hospitals)). Second, some essential variables recover their significance, such as household size, the proportion of wage earners, health insurance by occupational syndicates, and outpatient healthcare services. The most striking difference between the two models was that the total household expenditure was not significant in the homoscedastic probit model, whereas it was highly significant in both mean and latent scale models (heteroskedastic probit model), indicating that the inference based on the ordinary probit model is inaccurate in case of violation of the homoscedasticity assumption. Moreover, household size has a reversal sign and becomes significant after accounting for heteroskedasticity.

While the two models differ in terms of the number of significant variables, parameter estimates, and standard errors, some variables remain significant with the same sign in both models, such as the proportions of female, elderly, and sick members, which are positively associated with CHE. Moreover, based on the estimates of the two models, households headed by an illiterate individual, those headed by a man, those located in rural Lower Egypt, and those with chronically ill members suffer from CHE. Meanwhile, households headed by an educated, employed, and insured person are protected against CHE in both models. The two models demonstrated that age and age squared of household heads have no significant effects on the likelihood of incurring CHE. Also, the proportions of children, insured children, and insured elderly were not significant variables in both models.

Marginal effects and their standard errors are derived from both models (Table 1). Based on the estimated marginal effects of the heteroskedastic probit model, the probability of incurring CHE increases by 0.06 and 0.04 in male- and illiterate-headed households, respectively, whereas it decreases by 0.05, 0.09, and 0.08 if the household head has a secondary degree, a university degree, and a postgraduate degree, respectively. The employment status of the household head has a positive effect on the probability of CHE: it decreases the probability of CHE by 0.07. An increase in the proportion of educated members and wage earners in the household reduces the probability of CHE by 0.33 and 0.27, respectively. Meanwhile, the proportions of female, elderly, and sick members increase the likelihood of incurring CHE by 0.08, 0.03, and 0.06, respectively. The presence of chronic diseases raises the probability of CHE by 0.1, whereas the probability drops by 0.04 in households with health insurance coverage. Using private sector services greatly increases the probability of CHE by 0.39. The heteroskedasticity probit model showed that the infinitesimal change in total household expenditure reduces the likelihood of CHE by 0.07.

## Discussion

Household characteristics have a significant effect on health expenditure and the probability of encountering CHE. Poor households have the highest incidence rate of CHE; the distribution of CHE is regressive across economic quintiles. This is attributed to poor households’ inability to afford healthcare. As commonly observed in the literature [18], consumption necessities, such as food and shelter, exhaust the main bulk of a poor household’s budget, leaving a small share to healthcare. Meanwhile, better-off households could finance health payments and are more protected against CHE than poor households.

Our empirical results demonstrated that a large-size household is more likely to incur CHE. This is consistent with the findings of [19], who have suggested that the large size of households increases the probability of at least one member getting sick, especially in the presence of overcrowding and infectious diseases. Besides, the limited economies of scale in using medical care compared with other consumption items increase health payments. However, some studies have found that having a larger size protects households from CHE as it creates an opportunity for accumulating earnings [18, 20–22].

The high proportion of wage earners significantly protects rural households from CHE. It is an indicator of a household’s ability to pool financial resources and informative about health insurance coverage, especially if the wage earners are officially employed and receive regular income [23, 24]. This finding is in contrast to those of previous studies [25], which have revealed that a high proportion of wage earners is positively associated with CHE as they are more likely to seek healthcare and provide financial support to other household members.

The household composition reflects its health needs and required expenditure [26]. The results demonstrated that households with a high proportion of elderly members are highly likely to face CHE than those with high proportions of children. This is mainly due to their well-known health needs and their inability to work [7, 21, 22, 27, 28]; besides, they are more expected to face several episodes of illness [29]. In addition, female proportion in households is also positively associated with CHE. One key finding was that educated household members successfully shelter their households from CHE. This effect could be explained by the significance of education in reducing the anxiety associated with sickness and preventing making an irrational decision.

Household standard of living determines the amount of affordable health payments and the risk of facing CHE. The results revealed that the total household expenditure is negatively associated with the risk of incurring CHE. This association has been established by many studies [2, 21, 27, 30]. However, some studies have stated that the likelihood of incurring CHE decreases in response to an increase in household expenditure as poor households are forced to forgo or delay seeking healthcare to avoid fees, whereas better-off households are at higher risk of CHE because they have high responsiveness to health needs, and simultaneously, they prefer the highest quality private healthcare facilities [7, 19, 22, 24, 31].

Illness is the main driver of health expenditures and pushes households to reallocate considerable shares of their resources to OOP health payments, whereas demographic–economic characteristics control the burden of these expenditures. Our findings corroborate earlier literature that the presence of chronic diseases significantly increases the risk of incurring CHE [21, 27, 28, 32]. Meanwhile, the presence of disabilities does not have a significant effect on the risk of facing CHE, which contradicts the findings of [18, 33].

In the absence of health insurance coverage, health expenditures can absorb a substantial fraction of the household resources and severely disrupt living standards [33]. Health insurance coverage significantly reduces the chance of incurring CHE. A similar conclusion was reached by many studies. For example, Reddy (2013) has found that health insurance is negatively correlated with the risk of incurring CHE and indebtedness in three Asia-Pacific countries: China, Malaysia, and the Philippines [34]. Also, Van Minh and Tran (2012) have indicated that health insurance has a modest positive effect on CHE. Contrary to the widely held hypothesis about the role of health insurance in reducing financial hardships [21], some studies have shown that that health insurance has a contradictory effect on health payments: health insurance caused larger health payments as it facilitates access to healthcare services creating an induced demand for services [4, 18, 27, 35].

Our most intriguing finding is that increasing the proportion of insured children and elderly members had insignificant effects on the incidence of CHE. These findings support that increasing health insurance coverage does not necessarily reduce OOP health spending and achieve effective protection against CHE. For example, Iran has undertaken promising steps to expand health insurance coverage for most rural households, and 90% of the population has access to primary healthcare services; however, the incidence rate of CHE has been higher among insured rural households than uninsured ones. In addition, 49.2% of Iranian households had incurred debts, 21.7% sold their jewelry, and 15.9% used their savings to finance health costs [36]. These findings confirm that the provision of health insurance should coincide with adequate financial coverage for all required health needs, in addition to providing accessible health services of acceptable quality to deter recourse to the private sector.

To control potential confounders related to health insurance status, we used the available data about the type of health insurance. Although the selection of health insurance providers may depend on unobservable covariates [37], our analysis did not have this issue as the choice of health insurance in Egypt is highly restricted. There is no adverse selection as public sector employees and students are covered semi-compulsorily by Egypt’s primary insurance provider) HIO hospitals), and employees of other operational bodies are covered by occupational syndicates, whereas private health insurance is less prevalent in Egypt, particularly rural areas. Moreover, our model incorporated the measures of health status as the presence of chronic diseases or disability and the socioeconomic covariates to control for any selection effect.

The type of health insurance does not have a significant effect on protecting insured households from financial hardships, except that provided by occupational syndicates which, in contrast, is positively associated with CHEs. Some studies have shown that health insurance coverage has contrasting effects according to the benefits package design [18, 23]. Limited insurance packages with low depth of coverage, high co-payment rates, and relatively low-reimbursement ceilings increase the risk of CHE. For example, Shahrawat and Rao [38] have revealed that Indian insurance schemes with limited coverage (hospital expenses only) did not contribute to reducing CHE as outpatient care expenses occupied the main share of health expenditure. Kimani et al. [39] also have explained that health insurance fails to protect households due to its limited coverage of only bed costs for inpatient stays. Others have demonstrated that private health insurance offering a broad benefits package succeeds in protecting households from CHE compared with social insurance schemes [29].

Another factor influencing the likelihood of facing CHE is the healthcare provider; households who receive health services from private hospitals are most likely to afford CHE. It is worth noting that most OOP health payments go to the private sector. Although more than 90% of insured households are covered by HIO hospitals, only 19% use them, whereas 67.8% use private hospitals. This could be because the private health system offers better amenities, lower waiting time, and varied and high-quality healthcare services. It is quite a surprise to find that households using HIO or public hospitals have CHE.

Inpatient services resulting from unforeseen health shocks could be sizable and exhaust more resources in the short term, whereas, in the long term, outpatient services capture a relatively great amount of health payments in addition to drugs, especially for patients with chronic diseases. Our estimates corroborate the findings of previous studies that households using inpatient health services contribute to the increase in the risk of incurring CHE, especially those who sought treatment in private hospitals [29].

Regarding the characteristics of the household head, the likelihood of facing CHE is anticipated to increase as the age of the household head increases due to age-related health needs [18, 20, 23], the age of the household head and its squared value are not significant variables in our model. Also, in contrast to earlier findings that female-headed households are at higher risk of CHE [18, 28, 29], we detected that male-headed households are more likely to face CHE than those headed by females. Our estimates emphasized that households with educated heads are less likely to encounter CHE than those with illiterate heads. Educated heads consciously invest in health without making irrational decisions or incurring futile payments. In line with other studies, in this study, the employment status of the household head serves as a protective tool against financial catastrophes [18, 28, 29].

Although reducing poverty rates has a high priority on the development agenda of national governments and international agencies, several studies have focused only on poverty estimates in Egypt, whereas scarce empirical studies shave investigated the burdensome of OOP health payments and their impoverishing impact at the household level, which provides little evidence on the incidence of CHE and their determinants [12]. There are several notable strengths of this study. This study determined the underlying factors associated with the risk of facing CHE in rural areas that can provide the basis for analyzing policy options to alleviate the burden of catastrophic payments. Moreover, it explains the role of existing health insurance schemes in providing financial protection in rural areas. The key strength of this study lies in obtaining unbiased and consistent estimates, and declaring the implications of ignoring heteroskedasticity by highlighting the dramatic differences in marginal effects estimates between the ordinary and heteroskedastic probit models.

### Limitations of the the study

It is worth mentioning the limitations of the study. First, the households that are unable to finance health costs and do not seek healthcare were missed during analysis. This could result in underestimating the proportion of households that incurred CHE [26]. However, our overall estimates are not at risk because the percentage of households with zero health payments did not exceed 3% of the total sample. Second, the lack of longitudinal data is a major limitation as in other studies, longitudinal data were most suited to adequately capture the effects of health payments and make a causal analysis. Third, we cannot explore the other impoverishing effects of OOP health payments, such as using savings, incurring debts, and depleting productive assets as the survey did not include these effects.

However, this study revealed the influential predictors of incurring CHE. CHE is also related to the performance of the health system and its health insurance tools. CHE and the resultant impoverishment have been widespread in countries with a poorly functioning national health system, inadequate social welfare schemes, and poor economic performance. The characteristics of health services can exacerbate the financial burdens of illness; availability of health services at unlimited costs and low coverage can deter the poor from using health services or cause regressive burdens [10]. Therefore, further investigation is necessary to detect the role of the health system in shaping CHE.

## Conclusion

Heavy reliance on OOP health payments causes financial burdens for households and creates inequitable access to healthcare services, particularly in light of the growing presence of profit-seeking healthcare providers. CHE has received considerable attention in many countries and have been substantiated as a significant additional determinant of poverty; however, to date, the concern on CHE has remained insufficient in Egypt. OOP health payments remain the principal source of healthcare financing in Egypt as in most low-income countries. Identifying the characteristics that push households to be more vulnerable to CHE is of great importance for designing efficient health systems. In this context, this study was conducted to produce conclusions to help policymakers in conducting meaningful reforms in rural areas based on the incidence rates of CHE and their influencing factors.

Investigating the determinants of CHE in rural areas using a heteroskedastic probit model demonstrated that inference based on an ordinary probit model is inaccurate due to the violation of the homoscedasticity assumption. In the heteroskedastic probit model, larger households positively are associated with CHE, and having high proportions of elderly, sick, and female members positively contribute to the probability of incurring CHE. Moreover, having high proportions of educated members, wage earners, and insured members helps rural households escape from CHEs, whereas households with less educated, unemployed, and male heads exhibit higher probabilities of incurring CHE.

CHEs are substantially attributed to the increased demand of rural households for private sector services and little use of health services provided by public hospitals. Even Egypt’s primary insurance provider does not have a significant effect in protecting households against CHE. Using health services provided by HIO hospitals or public hospitals in rural areas increases the likelihood of encountering CHE.

Many policies could be developed from our findings, such as enhancing the role of social health insurance in rural areas and expanding the health coverage for chronically ill household heads to reduce the burden from their shoulders and protect them from falling into poverty. There is also an urgent need to limit and control OOP health payments absorbed by the private sector to achieve an acceptable level of fairness in financing. Expanding the scope of financial protection has become a priority in rural areas to lessen the incidence of CHE while ensuring access to healthcare services for disadvantaged groups.

## Data Availability

The data that support the findings of this study are available from Central Agency for Public Mobilization and Statistics (CAMPAS), but restrictions apply to the availability of these data, which were used under license for the current study, and so are not publicly available.

## Abbreviations

OOP health expenditure: Out-of-pocket health expenditure
CHE: Catastrophic health expenditure
HIECS: Household Income, Expenditure and Consumption Survey
CAPMAS: Central Agency for Public Mobilization and Statistics
HH Head: Household head
NHA: National health accounts
QMLE: Quasi maximum likelihood estimator
*ctp*_*h*_: Capacity to pay
*se*_*h*_: Food subsistence spending
